# Mitochondrial COI and morphological evidence for host specificity of the black cherry aphids *Myzus cerasi* (Fabricius, 1775) collected from different cherry tree species in Europe (Hemiptera, Aphididae)

**DOI:** 10.3897/zookeys.388.7034

**Published:** 2014-03-12

**Authors:** Rimantas Rakauskas, Jekaterina Havelka, Audrius Zaremba, Rasa Bernotienė

**Affiliations:** 1 Department of Zoology, Vilnius University, M. K. Čiurlionio 21/27, LT-03101, Vilnius, Lithuania

**Keywords:** Molecular systematics, cherry aphids, morphological key to subspecies

## Abstract

Partial sequences of the mitochondrial COI gene of forty eight European and two Turkish population samples of *Myzus cerasi* from different winter hosts (*Prunus* spp.) were subjected to phylogenetic analyses. The analysed *M. cerasi* samples emerged as paraphyletic relative to a *Myzus borealis* sample used as an out-group, and formed two major clades in neighbor joining, maximum parsimony, maximum likelihood and Bayesian inference trees, corresponding to subspecies living specifically on *Prunus avium* and *P. cerasus*. Multivariate discriminant analysis (method of canonical variates) was applied to find out if morphological variation of samples correlated with mitochondrial COI and host plant information. Mean scores on the first two canonical variables clustered samples fully in accordance with their COI haplotypes and host plants confirming the existence of two morphologically similar winter host - specific subspecies of *M. cerasi* in Europe. No single morphological character enabled satisfactory discrimination between apterous viviparous females of the two subspecies. A three-character linear discriminant function enabled 92.37% correct identification of apterous viviparous females of *M. cerasi cerasi* (n = 118) and 93.64% of *M. cerasi pruniavium* (n = 110). A key for the morphological identification of the two subspecies is presented and their taxonomic status is discussed.

## Introduction

Black cherry aphid *Myzus cerasi* (Fabricius, 1775) is reported to be a serious pest of cherries all over the world (Barbagallo et al. 1997, Blackman and Eastop 2000, Holman 2009) and its morphology, life cycle, host specificity and potential harmfulness have therefore been the subject of intensive studies (Ross 1918, Wimshurst 1925, Pokrovskyj 1932, Vereshchagina 1966, Fomicheva 1967, Karczewska 1970, Rakauskas 1984, Gruppe 1990, Cichocka and Goszczynski 2004). Nevertheless, the species level classification of black cherry aphids has not been satisfactorily resolved. The black shiny aphids inhabiting cherry trees were originally described as a single species, *Myzus cerasi* (Fabricius, 1775), but European populations inhabiting sweet cherry (*Prunus avium*) were later separated as *Myzus pruniavium* Börner, 1926. Börner’s species has been accepted by some (Börner 1943, 1952, Heinze 1961), but synonymised with *cerasi* by others (Miyazaki 1971, Eastop and Hille Ris Lambers 1976, Remaudière and Remaudière 1997), while others have treated it as a subspecies of *cerasi* (Shaposhnikov 1972, Favret 2014). Differences in host specificity of the two taxa have been documented in experimental transfer studies (Karczewska 1970, Dahl 1968, Vereshchagina 1966), showing *Myzus cerasi cerasi* as heteroecious, alternating between cherries (both *Prunus cerasus* and *Prunus avium*) and herbaceous hosts (*Galium*, *Euphrasia*, *Odontites*, *Veronica*). *Myzus cerasi pruniavium* Börner, 1926 differs from the nominative subspecies in having *Prunus avium* as the only winter host (it is incapable of living on *Prunus cerasus*), and also has a somewhat different summer host list. Enzyme electrophoretic studies indicated a reduced gene exchange between the subspecies (Gruppe 1988a), perhaps due to differences in the phloem sap of sour and sweet cherry causing divergent selection (Gruppe 1989). Morphological characters for discrimination between the two taxa have been suggested (Heinze 1961), but none of these enabled satisfactory separation between viviparous females of *Myzus cerasi cerasi* and *Myzus cerasi pruniavium* when applied to independent aphid material (Dahl 1968, Gruppe 1988b). The taxonomic status of the black cherry aphid in the western Palaearctic thus remains unclear (Blackman and Eastop 2000, 2006, Lampel and Meier 2007, Holman 2009, Osiadacz and Halaj 2010). They are possibly members of a complex of cryptic aphid species that includes *Myzus cerasi umefoliae* (Shinji, 1924), which overwinters on *Prunus mume* in Japan with *Artemisia* as its summer host (Miyazaki 1971). In northern Europe there is also another potentially distinct taxon that lives all year without host alternation on herbaceous plants (Dahl 1968), and to which the name *Myzus cerasi veronicae* (Walker, 1848) may be applicable.

A similar species complex is found in the mealy plum aphid, *Hyalopterus* spp. (Lozier et al. 2008). Separation of three *Hyalopterus* species was eventually justified by their distinctness at molecular level (Lozier et al. 2008), but they still remain difficult to separate by their morphological characters (Basky and Szalay-Marszό 1987, Blackman and Eastop 1994, 2000, 2006), including those used in the most recent identification keys (Lozier et al. 2008, Rakauskas et al. 2013). A similar recent case is that of birch- and oak-inhabiting aphid species of the genus *Stomaphis* Walker, 1870 (Depa et al. 2012).

The *Myzus cerasi* complex has not yet been the subject of detailed molecular study, although certain DNA sequences have been isolated (Foottit et al. 2008, Valenzuela et al. 2007, Clements et al. 2000, 2000a). Preliminary data on the partial sequences of the mitochondrial COI gene support the existence of subspecies of *Myzus cerasi* (Voronova et al. 2011). The aim of this study was to clarify the taxonomic status of the host-alternating taxa in the *Myzus cerasi* complex by a combined study of partial sequences of the mitochondrial COI gene and morphological characters of the European samples collected from different species of cherries.

## Material and methods

### Material studied

Fifty population samples of apterous viviparae of black cherry aphids from nine European countries and Turkey were collected in 2004–2013, mostly from *Prunus cerasus*, *Prunus avium*, but also from three other *Prunus* species (Table 1). Twenty samples were used for morphology - based stepwise discriminant analysis. The remaining 30 samples were used for subsequent evaluation of the derived discrimination functions. Samples of *Myzus borealis* Ossiannilsson, 1959 and *Myzus persicae* (Sulzer, 1776) (GenBank Accession No AB506741) were used as out-groups for the phylogenetic analyses.

**Table 1.** Aphid material used in the present study. Samples used for morphology-based discriminant analysis are given in bold.

Place, date, collection number; (number of individual apterae per sample)	GenBank Accession No
***Myzus cerasi* on *Prunus cerasus* L.**	
**Jieznas, Prienai distr., Lithuania, 2012.05.30, 12-25; (8)**	KF754311
Alytus, Lithuania, 2012.05.30, 12-30; (8)	KF754310
**Daugai, Alytus distr., Lithuania, 2012.05.30, 12-32; (8)**	KF754303
**Skirgiškės, Vilnius distr., Lithuania, 2012.06.05, 12-37; (8)**	KF754304
**Eišiškės, Šalčininkai distr., Lithuania, 2012.06.13, 12-43; (7)**	KF754329
**Labanoras, Švenčionys distr., Lithuania, 2012.06.19, 12-70; (8)**	KF754306
Molėtai, Lithuania, 2012.06.19, 12-72; (8)	KF754314
Kraujaliai, Molėtai distr., Lithuania, 2012.07.10, 12-120; (8)	KF754305
Žičkai, Molėtai distr., Lithuania, 2012.07.13, 12-132; (8)	KF754321
**Nida, Neringa, Lithuania, 2012.08.09, 12-176; (8)**	KF754322
**Smiltynė, Klaipėda, Lithuania, 2012.08.12, 12-191; (8)**	KF754309
**Preila, Neringa, Lithuania, 2012.08.13, 12-203; (8)**	KF754330
Bagnolo Mella, Brescia prov., Italy, 2013.05.01, 13-27; (8)	KF754338
Poncarale, Brescia prov., Italy, 2013.05.02, 13-33; (8)	KF754337
Suginčiai, Molėtai distr., Lithuania, 2013.06.15, 13-83; (8)	KF754345
Akmeniai, Lazdijai distr., Lithuania, 2013.05.30, 13-57; (8)	KF754341
Karsava, Ludza distr., Latvia, 2013.07.17, 13-133; (8)	KF754344
Gorodok, Vitebsk distr., Belarus, 2008.06.17, 08-6; (6)	KF754302
Zadrachje, Vitebsk distr., Belarus, 2008.06.18, 08-18; (8)	KF754325
Riga, Latvia, 2008.07.03, 08-73; (8)	KF754328
**Skirgiškės, Vilnius distr., Lithuania, 2011.06.15, 11-46; (8)**	KF754316
**Cluj Gilau, Romania, 2012.06.19, Z12-112; (8)**	KF754327
Cluj Gilau, Romania, 2012.06.19, Z12-116; (8)	KF754319
Poncarale, Brescia prov., Italy, 2013.05.02, 13-30; (8)	KF754346
Mezöpeterd, Hajdu-Bihar distr., Hungary, 2012.06.20, Z12-122; (8)	KF754333
***Myzus cerasi* on *Prunus avium* L.**	
Skirgiškės, Vilnius distr., Lithuania, 2012.06.05, 12-39; (7)	KF754332
**Šalčininkai, Lithuania, 2012.06.13, 12-48; (6)**	KF754317
**Bratoniškės, Vilnius distr., Lithuania, 2012.06.14, 12-56; (7)**	KF754313
**Blagoevgrad, Bulgaria, 2012.06.26, 12-83; (7)**	KF754318
Frankfurt/Main, Germany, 2012.06.30, 12-104; (8)	KF754307
**Kraujaliai, Molėtai distr., Lithuania, 2012.07.10, 12-111; (8)**	KF754308
**Stirniai, Molėtai distr., Lithuania, 2012.07.12, 12-128; (8)**	KF754320
**Juodkrantė, Neringa, Lithuania, 2012.08.10, 12-182; (8)**	KF754323
**Pervalka, Neringa, Lithuania, 2012.08.11, 12-188; (7)**	KF754336
Preila, Neringa, Lithuania, 2012.08.13, 12-199; (7)	KF754335
Rondo, Katowice, Poland, 2011.05.13, 11-10; (8)	KF754349
Tekir, Karamanmarash distr., Turkey, 2011.05.21, 11-25; (5)	KF754312
Göksun, Karamanmarash distr., Turkey, 2011.05.21, 11-27; (8)	KF754315
**Kairėnai, Vilnius, Lithuania, 2010.07.01, 10-3; (7)**	KF754324
Zafferana, Catania, Italy, 2004.06.28, 04-49; (5)	KF754339
Costinesti, Romania, 2012.06.15, Z12-90; (8)	KF754326
**Galata, Varna, Bulgaria, 2012.06.18, Z12-102; (7)**	KF754351
**Burgas, Bulgaria, 2012.06.19, Z12-110; (8)**	KF754331
Cluj Gilau, Romania, 2012.06.19, Z12-117; (7)	KF747679
Carpendolo, Brescia prov., Italy, 2013.04.27, 13-12; (7)	KF754340
Akmeniai, Lazdijai distr., Lithuania, 2013.05.30, 13-60; (8)	KF754347
Wojslawice, Lower Silesia, Poland, 2013.06.20, 13-98; (8)	KF754342
***Myzus cerasi* on *Prunus serrulata* Lindl.**	
Wojslawice, Lower Silesia, Poland, 2013.06.20, 13-97; (8)	KF754348
***Myzus cerasi* on *Prunus maackii* Rupr.**	
Dobele, Latvia, 2013.07.03, 13-119; (8)	KF754343
***Myzus cerasi* on *Prunus mahaleb* L.**	
Medias, Sibiu distr., Romania, 2012.06.19, Z12-113; (8)	KF754334
***Myzus borealis* on *Galium rubioides* L.**	
Zmejinyje ostrova, Kanev distr., Cherkasy reg., Ukraine, 2006.06.16, 06-74	KF754350

### DNA extraction, PCR amplification and sequencing

For molecular analysis, a single aphid individual from one sampled plant was considered as a unique sample. Total genomic DNA was extracted from a single aphid using the DNeasy Blood & Tissue kit (Qiagen), which involved at least a 2 h digestion of tissue with proteinase K. Partial sequences of mitochondrial COI gene were PCR-amplified using earlier published primers (Turčinavičienė et al. 2006). PCR amplification was carried out in a thermal cycler (Eppendorf) in 50 µl volumes containing 2 µl genomic DNA, 5 µl of each primer (10 µM), 5 µl of PCR-reaction buffer, 5 µl of dNTP mix (2 mM each), 4–8 µl of 25 mM MgCl_2_ and 1.25 U of AmpliTaq Gold 360 polymerase (5 U/µl) and ddH_2_O to 50 µl. The cycling parameters were as follows: denaturizing at 95 °C for 10 min (1 cycle), denaturizing at 95 °C for 30”, annealing at 49 °C for 30” and extension at 72 °C for 30” (37 cycles in total), and a final extension for 5 min (1 cycle). PCR products were subjected to electrophoresis on 2% TopVision agarose (Fermentas, Lithuania), stained with GelRed and sized against a MassRuler Low Range DNA ladder (Fermentas, Lithuania) under UV light. PCR products were purified and sequenced at Macrogen Europe (Amsterdam, the Netherlands) and Institute of Biotechnology of the Vilnius University (Vilnius, Lithuania). The amplification primers were also used as sequencing primers. DNA sequences for each specimen were confirmed with both sense and anti-sense strands and aligned in the BioEdit Sequence Alignment Editor (Hall 1999). Partial sequences were tested for stop codons and none were found. The sequence data have been submitted to the GenBank, accession numbers are given in Table 1.

### Analysis of DNA sequences

In addition to the sequences from 50 samples of *Myzus cerasi*, COI sequences of *Myzus borealis* from subgenus *Myzus* sensu stricto (the same subgenus as *Myzus cerasi*) and *Myzus persicae* from subgenus *Nectarosiphon* Schouteden, 1901 were selected as out-groups for the phylogenetic analyses, which included neighbor joining (NJ), maximum parsimony (MP), maximum likelihood (ML) and Bayesian inference in phylogeny (BI). NJ, MP and ML analyses were performed using MEGA 5 (Tamura et al. 2011). For NJ analysis Kimura 2-parameter (K2P) model of base substitution was used. Bootstrap values for NJ, MP and ML trees were generated from 1000 replicates. For ML analysis Tamura 3-parameter model with Gamma distribution (T92+G) was selected by MEGA 5 model selection option (Tamura et al. 2011). Bayesian analysis was conducted in MrBayes 3.2.1 (Ronquist and Huelsenbeck 2003) using Hasegawa-Kishino-Yano model with Invariable sites and Gamma distribution (HKY+I+G), which was selected by jModeltest (Posada 2008). Four simultaneous chains, 3 heated and 1 “cold”, were run for 3 000 000 generations with tree sampling every 1000 generations. The topologies obtained by NJ, MP, ML and BI were similar, so only ML tree is shown with values of NJ/MP and ML/BI bootstrap support and posterior probabilities over 50% indicated above and below branches respectively. Statistical parsimony haplotype networks were constructed for samples of *Myzus cerasi* and *Myzus borealis* using TCS v1.21 (Clement et al. 2000).

### Morphological study and discrimination analysis

Samples representing different clades in the molecular tree and haplotype network were used for stepwise discriminant analysis followed by canonical analysis: 10 samples from sour cherry, *Prunus cerasus*, and 10 samples from sweet cherry, *Prunus avium* (shown in bold in Table 1).

Based on earlier taxonomic work (Heinze 1961, Dahl 1968), 19 metric (in mm) characters were studied: A2L – length of antennal segment 2; A3L – length of antennal segment 3; A4L – length of antennal segment 4; A5L – length of antennal segment 5; A6BL – length of basal part of antennal segment 6; A6TPL – length of terminal process of antennal segment 6; BL – body length (excluding cauda); Bwant3 – basal width of antennal segment 3; CL – length of cauda; CW – basal width of cauda; DT3L – length of the second segment of hind tarsus; F3L – length of hind femur; FF – depth of the frontal furrow; SL – length of siphunculus; T3L – length of hind tibia; URL – length of ultimate rostral segment; URW – basal width of ultimate rostral segment; VBSLmax – maximal length of the ventral body hairs; VBSLmin – minimal length of the ventral body hairs.

Measurements of the slide-mounted apterous viviparous females were performed by means of interactive measurement system Micro-Image (Olympus Optical Co. GmbH). STATISTICA 8 version software (Statsoft 2007) was exploited for data analysis. Pearson’s correlation coefficients were calculated to evaluate the correlation of morphometric characters with body length. Characters with strong (| r | ≥ 0.70) statistically significant (p < 0.05) correlation with body length were removed from the further analysis: BL (r = 1.00), F3L (r = 0.83), T3L (r = 0.82), A2L (r = 0.7), A3L (r = 0.75), A4L (r = 0.71), A5L (r = 0.7). The remaining 12 characters were used for the forward stepwise discriminant analysis followed by canonical analysis, with sample collection number as the grouping variable, thus excluding information about the host plant from the analysis. Mean canonical scores of the first two canonical variables were represented as bivariate scatter plots, in order to show any clustering of samples.

Morphological characters that contributed most to canonical discrimination functions were evaluated as having potential for separation of taxa. An identification key was constructed based on these discrimination functions. The key was then tested on the 30 aphid samples that had not been used in its construction (listed in normal font in Table 1).

## Results

### Partial sequences of mitochondrial COI gene

Fifty partial COI sequences of *Myzus cerasi* and one of *Myzus borealis* from 11 countries were included in analysis. The alignment contained 616 bases in the final set with three variable sites, all of which appeared parsimony informative. The average base composition was A = 34.0%, C = 12.7%, G = 12.3% and T = 41.0%. The overall transition/transversion ratio R = 1.221 for all sites.

**Figure 1.**
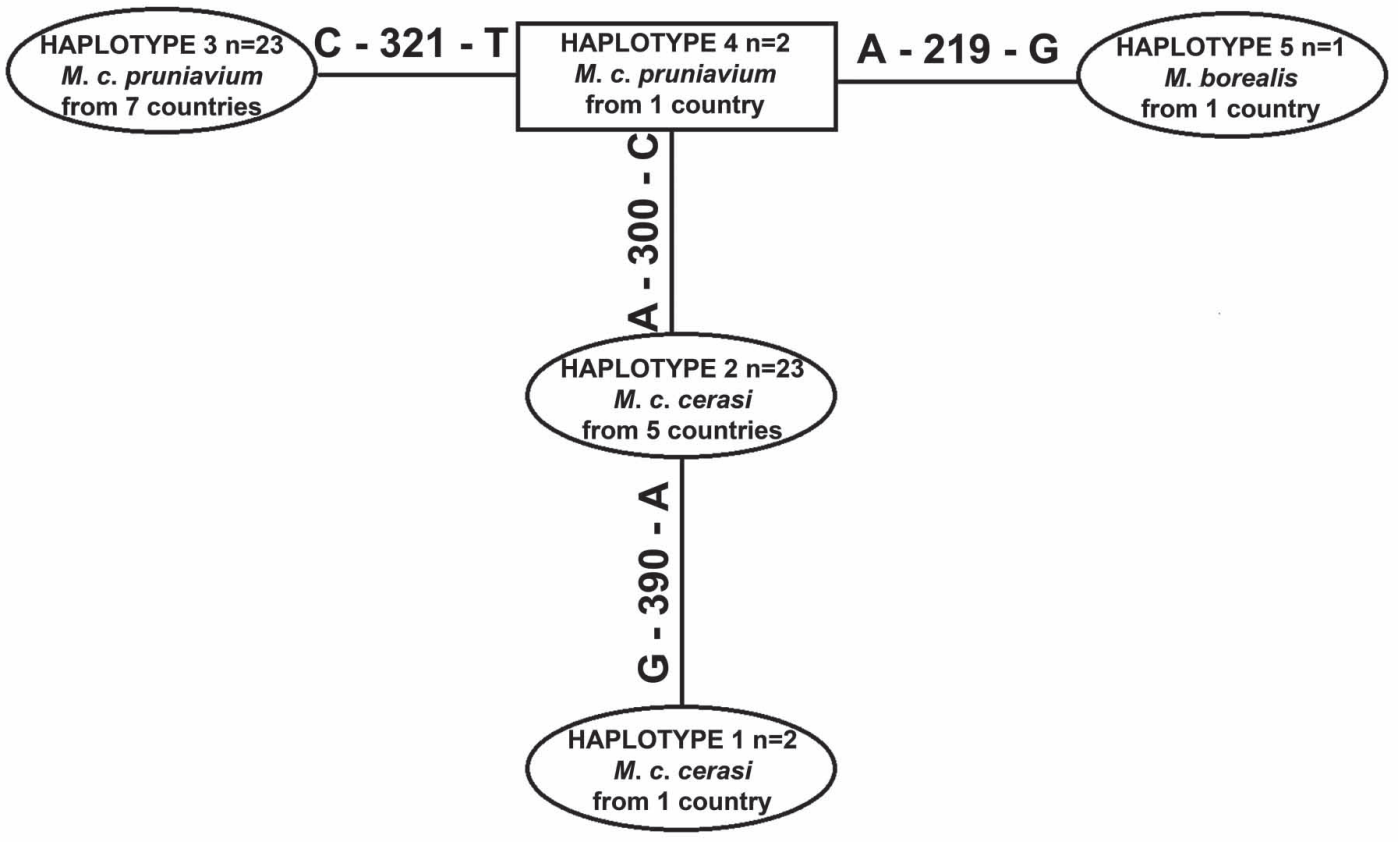
Haplotype network (TCS 1.21 software: Clement et al. 2000) for COI fragment (616 positions in final set) haplotypes of *Myzus cerasi* and *Myzus borealis*. The haplotype with the highest outgroup probability is displayed as a square, while others are displayed as ovals. For sample information, see Table 2.

Five COI haplotypes were detected (Fig. 1): one for *Myzus borealis*, two for samples from *Prunus cerasus* and two for samples from *Prunus avium* (Table 2). Aphids collected from *Prunus mahaleb*, *Prunus maackii* and *Prunus serrulata* had the same haplotype (No. 3) as the majority of samples from *Prunus avium*. COI haplotypes detected among samples from *Prunus cerasus* (No. 1 and 2) and the remaining *Prunus* species (No. 3 and 4) differed in the following nucleotide positions of 616 bp alignment: 300 (between No. 1–2 and No. 3–4), 321 (between No. 3 and No. 4) and 390 (between No. 1 and No. 2). The range of the intraspecific pairwise sample divergences (K2P model) was 0.0 – 0.5% (average 0.2%), whilst interspecific pairwise sample divergences between three species of *Myzus* ranged from 0.2 to 6.8% (Table 3).

**Table 2.** COI haplotypes of three *Myzus* taxa revealed by construction of haplotype network. Sample numbers are the same as given in Table 1.

Haplotype number	Number of sequences	Sequence length (bp)	Sample numbers
*Myzus cerasi cerasi* (collected from *Prunus cerasus*)			
1	2	616	13-33; 13-27.
2	23	616	08-6;12-32; 12-176; 12-30; 08-18; z12-122; 12-120; z12-112; 12-132; 12-25; 12-43; 12-191; 12-203; 12-70; 12-72; 08-73; 13-83; 13-133; 11-46; z12-116; 12-37; 13-30; 13-57
*Myzus cerasi pruniavium* (collected from *Prunus avium*, except where otherwise noted)			
3	23	616	11-10; 12-39; z12-110; 12-182; 12-104; 12-56; 12-83; 12-111; 12-199; z12-90; z12-102; 12-48; 12-188; z12-113 (*Prunus maackii*); 04-49; 12-128; 10-03; 13-60; 13-98; 13-97 (*Prunus serrulata*); 13-12; 13-119 (*Prunus mahaleb*); z12-117
4	2	616	11-27; 11-25
*Myzus borealis* (collected from *Galium rubioides*)			
5	1	616	06-74

**Table 3.** Range of pairwise interspecific sample divergences of mitochondrial COI gene fragment (K2P model) for three species of *Myzus* (number of samples used is in parentheses).

Species 1	Species 2	Range of divergence, %
*Myzus cerasi* (50)	*Myzus borealis* (1)	0.2 – 0.5
*Myzus cerasi* (50)	*Myzus persicae* (1)	6.6 – 6.8
*Myzus borealis* (1)	*Myzus persicae* (1)	6.8

**Figure 2.**
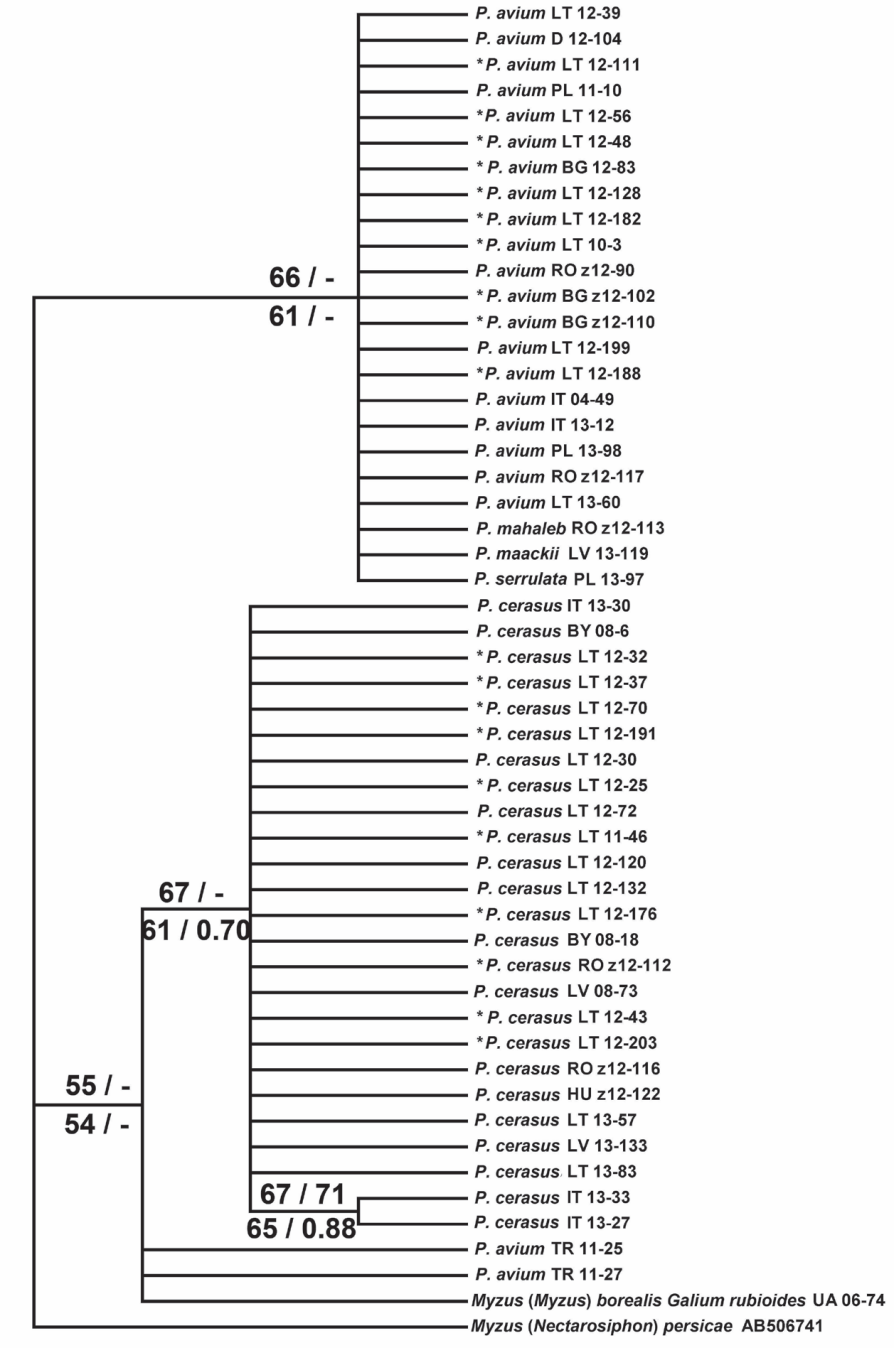
Maximum likelihood (ML) tree showing phylogenetic relationships among *Myzus cerasi* based on partial sequences of mitochondrial COI (616 positions in final set). Numbers above branches indicate support of NJ (left, > 50%) and MP (right, > 50%) bootstrap test with 1000 replicates, and numbers below branches indicate support of ML (left, > 50%) bootstrap test with 1000 replicates and posterior probabilities of BI analysis (right, > 0.50). Samples used for the discriminant analysis with *a priori* specified group membership followed by the construction of identification key are asterisked (*). The remaining samples were used for the *post hoc* classification. Sample numbers are the same as given in Table 1, together with the abbreviated symbol of respective country BG – Bulgaria, BY – Belarus, D – Germany, HU – Hungary, IT – Italy, LV – Latvia, LT – Lithuania, PL – Poland, RO – Romania, TR – Turkey, UA – Ukraine.

The maximum parsimony (MP) analysis of partial COI sequences resulted in 930 equally parsimonious trees (length = 43, CI = 1.00, RI = 1.00). The ML tree (T92 model) showed similar topology, as did NJ (K2P distances) and BI (HKY+I+G model) analyses. NJ, MP and ML bootstrap values over 50% together with BI posterior probabilities over 0.50 are given at respective nodes of the same tree in Fig. 2. Thus the *Myzus cerasi* samples form two major clades corresponding to two host-specific black cherry aphid taxa. One clade consists of all but two of the samples from *Prunus avium*, plus aphids collected from *Prunus mahaleb*, *Prunus maackii* and *Prunus serrulata*. The other clade contains all samples from *Prunus cerasus* and also includes the sample of *Myzus borealis* collected from *Galium rubioides*.

### Morphology

**Figure 3.**
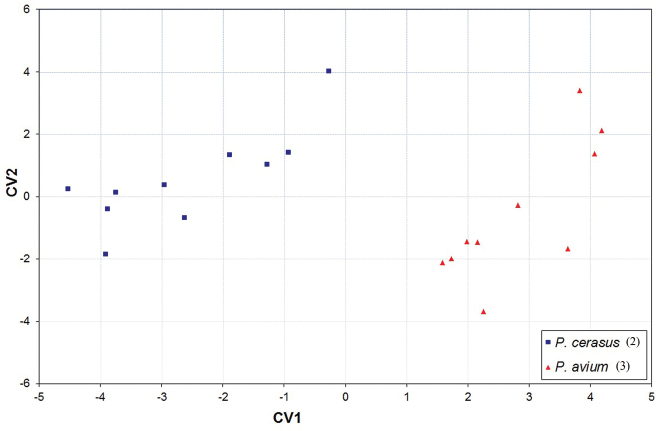
Plot of the mean scores of the first two canonical variates for 20 samples of *Myzus cerasi* (for specimen numbers per sample see Table 1). Samples cluster in accordance with winter host plant and COI haplotype (haplotype number is given in parentheses, see Table 2 for other haplotypes).

When morphometric data of apterous viviparous females from 20 different geographical localities were subjected to discriminant analysis with sample collection number as the grouping variable, the first two canonical variates (Fig. 3) clearly separated sour cherry samples (COI haplotype No. 2) from those collected from sweet cherry (COI haplotype No. 3). Length of terminal process of antennal segment 6 (A6TPL), length of siphunculus (SL) and maximal length of the ventral body hairs (VBSLmax) appeared to be important predictors for separation of the two taxa (Table 4).

**Table 4.** Contributions of 11 morphological characters to the canonical function discriminating 20 samples of *Myzus cerasi*. Character abbreviations are the same as in the text (Material and methods).

	Wilks’ Lambda	Partial Wilks’ Lambda	F-remove	p-level	Toler.	1-Toler. (R-Sqr.)
**A6TPL**	0.34	0.65	72.00	0.00	0.46	0.54
**SL**	0.30	0.73	49.26	0.00	0.31	0.69
**VBSLmax**	0.27	0.83	28.31	0.00	0.79	0.21
**Bwant3**	0.24	0.91	12.99	0.00	0.71	0.29
**URL**	0.23	0.93	9.51	0.00	0.65	0.35
**A6BL**	0.23	0.94	8.69	0.00	0.47	0.53
**DT3L**	0.23	0.97	4.62	0.01	0.49	0.51
**FF**	0.23	0.97	4.23	0.02	0.86	0.14
**VBSLmin**	0.23	0.97	3.70	0.03	0.85	0.15
**CL**	0.22	0.98	2.38	0.10	0.57	0.43
**CW**	0.22	0.99	1.75	0.18	0.56	0.44

**Figure 4.**
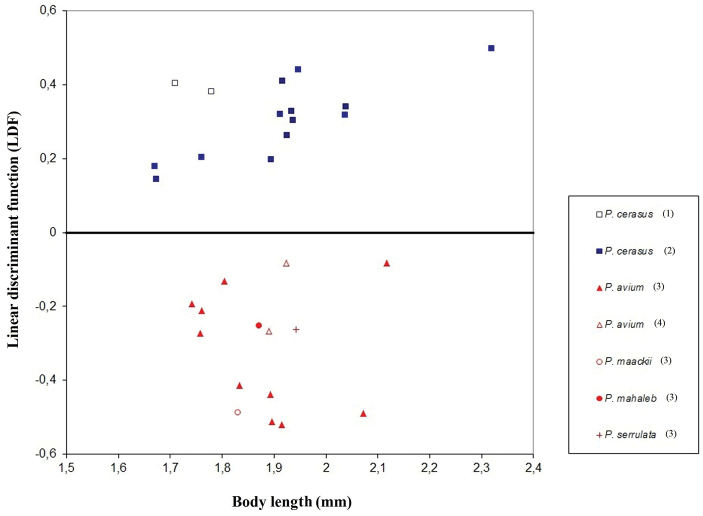
Plot of the mean scores of the individual LDF values (number of specimens per sample is given in Table 1) plotted against the mean body length for 30 samples of *Myzus cerasi* (normal font in Table 1) used to evaluate effectiveness of the eventual identification key. The icons are color-coded to match the COI haplotypes. Samples cluster in accordance with winter host plant and COI haplotype (haplotype number is given in parentheses, see Table 2 for haplotype information).

To discriminate between apterous viviparous females of host-specific black cherry aphid samples representing different clades in the haplotype network and the phylogenetic tree (Figs 1–2), the following linear discriminant function (LDF) was obtained: 3.924682 × SL - 5.6667 × A6TPL - 32.5504 × VBSLmax + 1. Using this LDF, 97.37% individuals from the whole dataset were reclassified correctly into their *a priori* specified groups with host plant species as grouping variable, including 96.2% of apterous viviparous females from *Prunus avium* (n = 79) and 98.6% from *Prunus cerasus* (n = 73). The *post hoc* classification of the remaining thirty samples gave 92.37% correct specimen identification of *Myzus cerasi cerasi* (n = 118) and 93.64% of *Myzus cerasi pruniavium* (n = 110). The scatterplot of the mean LDF and body length values calculated for each of 30 samples representing different host specific subspecies of *Myzus cerasi* is shown in Fig. 4. The following key is therefore suggested for the identification of apterous viviparous females of the two subspecies of *Myzus cerasi* when sampled from winter hosts.

### Key to European subspecies of *Myzus cerasi* on *Prunus* (apterous viviparous females)

**Table:** 

1	Value of LDF [3.92× (length of siphunculus) - 5. 67×(length of terminal process of antennal segment 6) - 32.55×(maximal length of the ventral body hairs) + 1] greater than zero. On *Prunus cerasus* (and sometimes on *Prunus avium*)	*Myzus cerasi cerasi*
–	Value of LDF less than zero. On *Prunus avium*, *Prunus maackii*, *Prunus mahaleb*, *Prunus serrulata*	*Myzus cerasi pruniavium*

## Discussion and conclusions

The combination of genetic distance evaluation with phylogenetic tree-building methods and multivariate analyses of morphometric data has been successfully applied to solve taxonomic problems in aphids, particularly in the genera *Hyalopterus* (Lozier et al. 2008), *Pentalonia* (Foottit et al. 2010), *Aulacorthum* and *Neoaulacorthum* (=*Pseudomegoura*) (Lee et al. 2011a). Based on the global data set, the average genetic divergence of COI barcode sequences between aphid species within the same genus was reported to be 5.84% (range 0.46 – 11.3%), and that within species 0.05% (0.00–1.00%) (Foottit et al. 2008, Lee et al. 2011b). Interspecific divergence of six species representing three subgenera of *Myzus* calculated for COI barcode sequences was reported as ranging from 5.55 to 11.3% (Foottit et al. 2008). In comparison, partial COI sequences (GenBank, 1145 bp) of three *Aphis fabae* subspecies (*Aphis fabae fabae*, GenBank accession numbers FJ965713, FJ965717–FJ965718; *Aphis fabae cirsiiacanthoidis*, FJ965698–FJ965709; *Aphis fabae mordvilkoi*, FJ965710–FJ965712) show values of genetic divergence (K2P model) ranging from 0.00 to 1.42% (Table 5). Therefore, the range of genetic divergence between the two clades of *Myzus cerasi* emerging in phylogenetic trees presented in this paper (0.0 to 0.5%) appears to be of intraspecific level. Based on the available COI data, black cherry aphids inhabiting sour and sweet cherries should therefore still be regarded as a single species.

**Table 5.** Pairwise sample divergences of 1145 bp mitochondrial COI gene fragment (K2P model) between three subspecies of *Aphis fabae* (number of sequences used is in parentheses).

Subspecies 1	Subspecies 2	Mean and range of divergence, %
*Aphis fabae fabae* (3)	*Aphis fabae cirsiiacanthoidis* (12)	1.2 (0.97–1.42)
*Aphis fabae fabae* (3)	*Aphis fabae mordvilkoi* (3)	0.15 (0.00–0.26)
*Aphis fabae cirsiiacanthoidis* (12)	*Aphis fabae mordvilkoi* (3)	1.05 (0.97–1.15)

*Myzus borealis* is clearly closely related to *Myzus cerasi* and differs by only 0.2–0.5% of the COI sequences involved in the analysis. This suggests that it may also belong to the same species level taxon. More samples of *Myzus borealis* are needed to confirm this hypothesis. However, it should be noted that partial COI sequences of two biologically distinct *Macrosiphum* species, *Macrosiphum rosae* (Linnaeus, 1758) and *Macrosiphum knautiae* Holman, 1972 are very similar (Turčinavičienė and Rakauskas 2009), and low divergence levels have also been reported for *Bursaphis* species (Rakauskas et al. 2011) and adelgids (Žurovcová et al. 2010). It seems probable that in rapidly speciating aphid groups one may expect to find low levels of COI sequence divergence between taxa that are nevertheless functioning as distinct species.
